# Surgery

**Published:** 1825-01-01

**Authors:** 


### SURGERY.

1. *Amputation of the Lower Jaw.** In the spring of 1821, Mary Rice fell on the pavement with much violence. She was taken up insensible, her chin severely contused, and the front incisors of the lower jaw loosened; these soon became discoloured and still less firm in their attachment, whilst she complained of pain, shooting along the throat, from the chin to both ears. In the course of three months, a small, hard, fleshy-looking tumour was observed under the apex of the tongue; and now the front part of the jaw began to enlarge downwards and forwards: although there was much pain and loss of sleep, the little patient retained her appetite and the power of mastication. In the early part of 1823, however, the parts became frightfully enlarged, the bone swelled downwards, and the tumour, beneath the tongue, rose above the level of the teeth, wholly preventing mastication. The disease spread rapidly, and on July 10th, 1823, Mr. M'Clellan was called in. The patient was six years old, her countenance wan and haggard, and body emaciated. The whole substance of the bone, in front of its angles, projected downwards before the neck, and backwards rather to the left side of the throat, attenuating the under lip, which was much extended. The skin was highly vascular. Her mouth was thrust open by a huge tumour, rising from the inner surface of the inferior maxilla, and protruding in the shape of an enlarged tongue. Above the molar teeth, it was applied to the roof of the mouth, overlapping, anteriorly, and including in its substance the incisors. Its posterior limits could not be ascertained. Nothing but fluids could be taken, and from the difficulty of deglutition and respiration, the tongue must have been driven almost into the pharynx. As the only chance of saving her life, an operation was decided on, and, on the 13th of July, it was performed in the following manner: An incision was made through the integuments, from the left commissure of the lip obliquely downwards and outwards, over the anterior edge of the sterno-mastoideus, so as to command the carotid, should it be necessary to tie it on that side. The anterior edge of this incision was then raised, and the lower part of the tumour exposed. The integuments were next dissected forwards, till the whole tumour was exposed round to the opposite side. The facial artery was at once secured, where it emerges from the submaxillary gland, when the hæmorrhage ceased from nearly all the divided twigs. The insertions of the masseter muscles were then dissected up, to expose the sound bone, which was immediately divided by a metacarpal saw. The tumour was then turned outwards from the mouth and dissected from the under surface of the tongue, the submaxillary glands, and the muscles on each side. Part of the sublingual glands and left submaxillary, being tumefied and rather discoloured, they were cut out. Only Three arterial twigs beneath the tongue required the ligature.

* Dr. M'Clellan. Medical Review and Analectic Journal, No 1.

six or eight ounces of blood were lost during the operation, which took up four minutes and a half. The patient did not faint, but was at first nearly suffocated with the blood which passed down her throat. It being ascertained that no morbid structure was left behind, the large flap was re-applied, and the edges of the external incision kept together by three interrupted sutures and adhesive plaisters. The cavity beneath the tongue was partly filled with patent lint, bent into the shape of the lost bone, upon which the hanging integuments were lightly braced by a bandage. After the dressing, the tongue regained its natural situation, and the little patient could articulate and even drink some water. No pain was felt and she slept soundly: the bowels were kept open, and broths, &c. given for food. On the 4th day, the dressings being removed, the external wound had agglutinated by inflammation; the internal cavity was suppurating profusely, and granulations having sprung up, the dressings were removed altogether, on the 7th day. In three weeks complete cicatrization externally and internally. The cut extremities of the bone had ossified to about an inch in front of the angles, and the new flesh beneath the tongue had become a ligamentous mass, which answered for bone, and to it the muscles became adherent. The child soon became robust and grew rapidly, with her articulation accurate. The only external trace of the operation was the linear cicatrix of the first incision and a transverse wrinkle beneath the chin, from the contraction of the integuments. About four months afterwards, however, she caught a cold with fever, and, soon, the right submaxillary and neighbouring lymphatic glands became swollen and painful. Under depletion, &c. all speedily subsided, except the submaxillary gland, which continued indurated, while the integuments of the chin became dry and corrugated. The cicatrix under the tongue assumed a yellowish-white colour, and the chin increased in density. In January, there was excessive tumefaction, with external ulcerations. Deep fissures formed in the skin, discovering a dense mass of a cheesy, cartilaginous nature, like the tumour which had been removed. Large sloughs penetrated the cheek, exposing the cavity of the mouth, with intolerable fœtor. Emaciation and debility rapidly hurried the little sufferer to the grave. She died on the 22nd of March. Little pain or loss of sleep had been experienced. On dissection, the heart was found enlarged in substance and much distended with blood. Two small cartilaginous incrustations on the surface of the ventricles. Pericardium fully distended with reddish serum. About one third of the exterior of the lungs was quite white, the remainder as usual. Some small tubercles, two scrofulous, about the ramifications of the left bronchia. No other viscus diseased but the liver, which was studded over with white spots, of the size of grains of wheat.

---

2. *Treatment of Herniæ**. Dr. Kinglake may bid adieu to the ho-

* Dr. Kinglake. Med. and Phys. Journal, Nov. 1824.
Dr. Balfour. Ditto ditto.

nour of becoming one of "the peerage of the profession,"—that is, a member of the "New Society of Physicians." He has "lost caste," by daring to pollute his fingers by contact with a "hernial protrusion." As long as the worthy doctor confined the application of cold water to gouty toes, he was safe:—but when he extended his favourite remedy to bubonocele, and transgressed the laws of the Society "*by pressing steadily yet forcibly at the basis of the swelling, with a view to insinuate, as it were, a small portion of the air lying nearest to the hernial aperture,*" he passed the Rubicon, and shut himself out from the "PEERAGE." Dr. Kinglake has occupied some pages of our respected cotemporary with the important discovery of employing *cold* to a "hernial protrusion"—a discovery which may be found in every surgical work for the last twenty or thirty years.* The rationale of the application is equally novel—namely, the influence of cold in condensing the rarified air of the hernia below the stricture, "and imparting to it a density or resisting power that renders it capable of being wielded and pressed with *a wedge-like force* against the abdominal aperture." To this last part of the rationale, we willingly assign the title of *novelty.* Perhaps Mr. Brande or Sir Humphrey may invent a frigorific mixture that will give *solidity* to air, and then its "wedge-like" *form* as well as *force* will be irresistible. There is another proposition in the doctor's erudite paper, to which the surgical world will, we are convinced, unanimously assent—namely, that—"to endeavour to force into the cavity of the abdomen, through a definite opening, *a bulk much too large for its dimensions,* is to grapple with an impossibility." Dr. Kinglake has forgotten to give us an explanation of the expansion or rarefaction of air on passing from a temperature of 92°, inside of the abdomen, to one considerably lower, when outside of that region. "In intestinal hernia," says the doctor, "the size is chiefly made up of *air*, the *rarefaction of which* causes a distending *expansion* that is often acutely painful." We see no cause for this expansion of air—and yet we allow that expansion of bulk in the hernial protrusion often takes place. Is this not owing to the extrication of a *gazeous* fluid from the contents of the bowel, in consequence of the diminished vitality in a portion of incarcerated gut? Do we not see the same thing take place in the intestines of people who are moribund—and still more strongly after death? This, we think, is the true cause:—not expansion of air, but extrication of gas. The application of cold is rational and proper—but who, except Dr. Kinglake, would think of bringing the practice forward, at this time of day, with all the "pomp and circumstance" of a novel improvement in hernial taxis.

Not so with our worthy friend of "Auld Reekie," Dr. Balfour. He has made another *striking* discovery—namely, that the best way of

---

* See Hooper's Surgeon's *Vade Mecum,* 3d edition, page 142—or rather Sir Astley Cooper's great work, from which the article *hernia* is entirely taken. We quote the *Vade Mecum* purposely on this point, that Dr. Kinglake may not have to go far for the novelty of his practice.

pushing *up* a hernial protrusion, is to pull it *down*. Has the doctor taken a trip in the steam boat to Belfast lately? Else where did he learn to make bulls? It was so long ago as 1814, that Dr. Balfour was so fortunate as to reduce an incarcerated hernia in the above novel manner, and, "strange to say, he entirely forgot the fact, till one day lately when rummaging among a parcel of manuscripts," the case presented itself to his eye. He lost no time in taking the opinion of the most eminent surgeons in Edinburgh, and it appears, that "every one stared at mention of the fact." "All agreed that it was new"—and all were astonished that the idea did not strike themselves long ago. The doctor has only produced one specific opinion, however, and that is in the form of a short note from Mr. Liston. His answer, in our humble opinion, is any thing but favourable. He too admits that the practice is new; but he "doubts the practicability in some instances and the propriety of it in others." Surely Mr. Liston must have been quizzing the doctor when he admits the "sound principles" of pulling down a piece of fresh intestine through the stricture, in order to give the inflamed and swelled part below time to cool and shrink! Such, however, is the principle on which this new practice is founded. We refer the reader to the journal in question for further particulars.

---

3. *Paracentesis Thoracis.** In the course of last winter, we recollect hearing read, in one of our medical societies, a proposal from Dr. Carson, of Liverpool, to make an opening into the chest, in cases of ulcerated lungs, or in other words, broken down tubercles, in order to let the lung collapse and the ulcer heal. We were astonished that any modern physician, acquainted with the general condition of the lungs in confirmed phthisis, should make such a proposal, or execute, as Dr. Carson did, so hazardous and hopeless an operation. We scarcely remember opening any case of suppurated tubercles where the surrounding portions of lung would have permitted collapse however large an opening might have been made into the chest. To this, may be added the adhesions which so generally prevail in such conditions of the respiratory apparatus. The very case in which Dr. Carson tried the experiment, proved the truth of the above observations. The following case will also bear on this subject, and may afford a salutary caution to the young and temerarious surgeon.

*Case.* A man of the name of Meyer, 42 years of age, ill for several years, had had a venereal affection, for which he took a great deal of mercury. There supervened a diarrhœa, which, after having resisted every kind of treatment, went off of itself. He was much addicted to drink. In the summer of 1819, he was attacked with pneumonia of a violent character. Leeches and blisters applied; for a long time he spat blood and was harrassed with cough and difficulty of breathing.—

---

* Dr. Wedemeyer. Mag. Fur die Gesammte Heilkunde.

These symptoms, however, disappeared, to usher in another kind of disease, accompanied with swelling of the extremities. At the same time, rheumatism developed itself in the arm, for which the Limmer waters were taken. About the end of August, 1820; the swelling of the feet and asthma acquired additional intensity; cough convulsive; urine scanty and sedimentous; countenance livid and cachectic, with slight diarrhœa. There never existed a well-marked fever; though the pulse was small and frequent. No pain in the chest, but a greater feeling of constraint in the left, than in the right side. In the affected side a seton was inserted, and digitalis, squills and infusion of Saint Ignatius' bean were ordered. These attacks went on augmenting, and the patient entered the hospital with the following symptoms:—great difficulty of breathing; dry and convulsive cough; lies on his back; cannot recline on either side; pulsations of the heart almost imperceptible; pulse quick, more than 100 in the minute by day, and upwards of 110 towards evening and by night. The right side of the chest, which appeared at first the seat of inflammation, gave a dull sound by percussion, which caused a suspicion of adhesions there. The right side sounded clear, but more so towards the spine—a feeling of oppression in the precordial region as though effusion had taken place above the diaphragm. The skin of the thorax, as it were, infiltrated, œdematous; countenance bloated and of a bluish hue. The lower extremities, the left one more than the right, considerably swollen; diarrhœa trifling;—often during the night he awoke starting, &c. The author and another surgeon suspecting effusion into the thorax, persuaded the patient to submit to paracentesis. An incision was made, about three inches in extent, between the sixth and seventh ribs, in the middle of the space comprised between the sternum and vertebral column, on the left side of the chest. No water issued out, but the lungs filled the opening; respiration became difficult and the disease acquired its utmost intensity. The finger was introduced, then a full sized sound, into the wound: no liquids appeared. Here, thinking the true state of the case had been mistaken, they stopped up the wound with sticking plaister. The difficulty of breathing augmented, inflammatory symptoms developed themselves, and, 48 hours after the operation, the patient died.

*Dissection.* Right lung perfectly sound and without adhesions. Two ounces of bloody serosity were inclosed in the thoracic cavity of the same side. Heart healthy, though the pericardium contained a little water. The left lung was neither altered nor inflamed. The wound went deep into the thoracic cavity;* the incision had been made too low and it is probable, says the author, that the diaphragm had been

---

* We cannot understand how they ascertained this circumstance, unless it was by observing a wound in the lung of that side. It surely was bad surgery to push a trochar to any depth, without being perfectly certain that a fluid was present opposite to the site of the puncture. The pleura ought to have been divided carefully with a scalpel, where there was any doubt.—*Rev.*

wounded. (It is singular, that in opening the body, this is expressed as a doubt.) The left cavity of the thorax contained about a pint and a half of a serous turbid liquid. The lung, which, in one place was adherent to the pleura, was found placed before the wound as was said above. The liver was much softened and adhered in many places to the peritoneum.

As was remarked at the beginning, this operation may prove a salutary warning to bold surgeons. Indeed, we cannot see the prospect of advantage in drawing off an effused fluid from the chest, which has resulted from inflammatory action. The cause must be removed, and the absorption trusted to nature, assisted by medicine rather than the knife.

---

4. *Tracheotomy performed with a successful result in Cynanche Trachealis.* In the Repository for July, a case of this kind is published by P. Hume, Esq. of Coventry. However easy the operation of tracheotomy in itself really is, it is generally considered as formidable, and sometimes, perhaps, the unwillingness of practitioners to undertake it may deprive the patient of the only remaining chance of escape from the formidable attacks of croup, &c. The subject of Mr. Hume's case was a female of the name of Boddington, aged 31, who suffered an attack of croup, but did not call in his assistance until it was too late to expect any benefit could be derived from medicines.

A consultation was immediately called, the patient was bled; and the operation performed in an hour after the first visit of the surgeon. She was relieved as soon as the trachea was cut into. She was put to bed in a sitting posture, the wound was neither dressed nor covered. A saline aperient was exhibited. In the evening of the same day, she was free from fever, pulse 95.

She continued to go on well, and in twenty days after the operation, was enabled to attend to her domestic concerns. Mr. Hume observes, "it is questionable how far the operation may be successful in children." That in young subjects the difficulty of operating will be increased must be admitted, but we apprehend the same good effects may be expected from it when performed in them, as in adults. The late Mr. Chevalier performed the operation in children, and succeeded we believe twice in rescuing his patients from the distress and probably from the destruction of croup. There are many cases on record in which the presence of foreign bodies, in the trachea, has rendered the operation necessary, and in which it has been performed with success. Each case in addition, however, must be considered valuable and worthy of notice.

---

5. *Extirpation of the Ovaria.** Passing over the records of surgery, all of which cannot be depended on, we shall come at once to the recent facts, or alleged facts, communicated in this paper. Three cases of

* Mr. Lizars. Ed. Journal, Oct. 1824.

ovarian extirpation occurred, it would seem, some years ago, in the practice of Dr. Macdowal, of Kentucky, which were transmitted to the late John Bell, and fell into the hands of Mr. Lizars. We candidly confess that we are rather sceptical respecting these statements, and we are rather surprized that Mr. Lizars himself should put implicit credence in them. A woman, supposed to be parturient, was visited by Dr. Macdowal, at the instigation of two physicians, who considered her in the last stage of pregnancy. Dr. M. found the uterus unimpregnated, but a large tumour in the abdomen moveable from side to side. The woman travelled sixty miles on horseback to have an operation performed. Dr. Mac. made an incision, nine inches in length, parallel with the rectus abdominis, and right into the abdominal cavity. The tumour appeared in view, but could not be removed. A ligature was thrown round the Fallopian tube—the tumour cut open (found to be the ovaria) and fifteen pints of dirty gelatinous stuff extracted—"after which we cut through the Fallopian tube, and extracted the sac, which weighed seven pounds and a half." As soon as the external opening was made, the intestines rushed out upon the table, and they could not be replaced till after the operation was performed, which lasted 25 minutes! The wound was sewn up by means of the interrupted suture, assisted by means of adhesive plaster. Dr. Mac, visited the patient *at the end of five days*, though!! she had come to his own residence to have the operation performed. He found her engaged in making her bed! she soon returned to her native place quite well. *Credat Judæus, non ego.* The second case is little less extraordinary, if not incredible. A negress had a hard, painful, fixed tumour in the abdomen. Dr. Mac, placed her on a table—laid the abdomen open—inserted his hand—and found the ovaria very much enlarged—painful to the touch—and firmly adhering to the bladder and fundus uteri. "To extract *this* (two ovaria) he thought would be instantly fatal;" "but, by way of *experiment*," says the doctor, "I plunged the scalpel into the diseased part, when some gelatinous substance, as in the above case, with a profusion of blood, rushed to the external opening, which I conveyed off by placing my hand under the tumour, suffering the discharge to run over it." A quart or more of blood escaped *into the abdomen*. The same dressings, and the same success as in the first case! We cannot bring ourselves to credit this statement. Not so with the operation performed by Mr. Lizars himself. It was of a very different description from those said to have happened in Kentucky.

A woman had, for some years, suffered from an enlargement of the abdomen; which had been often mistaken for pregnancy by medical practitioners. "On examination," says Mr. Lizars, "the tumour occupied the *whole* abdominal cavity, and appeared to roll *from side to side*." This is rather loose description, and we are somewhat at a loss to understand it. There was also a lumbar abscess in the case. She had been twice tapped for dropsy of the left ovary. She suffered great pain, and anxiously desired an operation, which was performed by Mr. L. on the 24th October, 1823, in presence of Drs. Campbell and Vallange, together with Mr. Bourchier, of the 36th Regiment. He commenced the operation with a longitudinal incision on the left side, parallel with the linea alba, and reaching from near the ensiform cartilage to the crista of the pubis, through the integuments. The peritoneum was then cautiously opened, and the internal made to correspond with the external incision. The intestines rolled out, and were enveloped in a warm and wetted cloth. Mr. Lizars now proceeded to examine the state of the tumour—but to the astonishment of all present there was no tumour to be found. We think it was very fortunate for the operator that this was the case—for had there been a tumour extirpated, the patient would have had little chance of life. She did recover from the present operation, however, and lives to tell the tale. The cause of the deception does not appear very clear to us. Mr. Lizars attributes it to "the great obesity and distended fulness of the intestines, together with some protrusion pubic of the spine at the lumbar vertebræ." Be that as it may, we do not think that the cases brought forward in this paper, will have the effect of rendering surgeons more bold in operating for the removal of abdominal tumours, whether ovarian or of any other kind.

---

6. *Chronic Enlargement of the Tonsils.** "Catherine Collins, ætat. 10, presented herself to me in the month of June last, with considerable enlargement of both tonsils, by which deglutition and articulation appeared much impeded, her countenance expressive of much anxiety and distress, respiration affected; she appeared emaciated; for some time back she has been unable to swallow any thing but fluids, and has been fed on broths. On particular enquiry into her case, I ascertained that about two years back she had had simple cynanche tonsillaris, which was then neglected and has since gradually verged into the chronic state, slowly increasing until I saw her. Nothing decisive previously had been done for her. On examination, I found the tumours of a pyriform shape terminating in apices, the bases apparently involving the substance of the tonsils themselves, occupying nearly the entire space of the half arches of the velum pendulum palati; the uvula hanging undisturbed in the centre; between them a fissure appeared capable of admitting a crow quill into the pharynx. Their surface appeared covered with venous ramifications turgid with blood; texture firm and compressible in some parts, indurated in others. I determined to try local treatment previous to the proposal of extirpation, as the patients' sufferings were such as loudly to call for decisive interference. I immediately made several deep incisions into their substance, conceiving that the local hæmorrhage would materially contribute to their reduction; purgatives of the saline class were liberally exhibited, and extensive irritation of the cutis produced by frictions of the ung. antim. tart. below the angles of the jaws, and corresponding as near as possible to the internal tumours.

* Dr. Wm. Belcher, Member of the Royal College of Surgeons, in Ireland. Bandon, County of Cork.—*Private Correspond.*

The pil. hyd. submuriat. c. was afterwards prescribed as an alterative. This method of treatment was repeated and assiduously persevered in for three months, and it was satisfactory to observe that the tumours were gradually reduced, to the great comfort of the patient, and are now nearly extinct. I relate this simple case merely for the purpose of corroborating the good effects of this truly valuable agent (the ung. ant. tart.) when persevered in. In my opinion, we owe much to the late illustrious and lamented Dr. Jenner, who first brought this remedy into practice, and called the attention of the profession to it in his letter to the late Dr. Parry, of Bath. If these methods had failed, I was determined to extirpate the tumours by the ligature in preference to the knife (by the silver wire applied with Levret's double cannula.) My reason for this preference is from witnessing a case of extirpation by a scalpel when I was a surgical pupil. The subsequent hæmorrhage was so profuse that the patient was nearly lost, and nothing but the immediate application of the actual cautery saved his life. The ligature to be sure is tedious and sometimes productive of irritation; but query? is it not the safer method of the two? We have great authority certainly for the knife. Desault used both, but he preferred the knife, as also do most modern surgeons, Dupuytren, Lisfranc, and Beclard, at Paris; Sir A. Cooper, and others, in London; Liston, in Edinburgh; and Colles and Carmichael, at Dublin, think of nothing else. I should like to hear the decided opinions of the most experienced on this subject."

---

7. *Sudden Death during an Operation.* A most melancholy event happened lately in the surgical practice of M. Dupuytren, which involves some curious physiological and surgical considerations. On the 19th November, 1822, a fine young woman (Alexandrine Poirier) came to the Hotel Dieu, for a tumour of some size, situated on the posterior and lateral part of the neck. From its hardness, renitency, and insensibility, M. Dupuytren ascertained that it was of a cellulo-fibrous nature, and proposed its removal, to which the young woman consented. The operation was performed on the 22d of November, with all the skill and dexterity of that celebrated surgeon. No arteries were cut that required the ligature, and consequently, there was very little hæmorrhage. Neither were there any muscles or large nerves divided. Just, however, as he was proceeding to separate the last shreds of attachment, and turn the tumour out, he was surprised to hear a somewhat prolonged hissing noise (sifflement prolongé) similar to that produced by the re-entrance of air into a vessel from which it had been exhausted. The operator stood for an instant astonished, and observed, that, were it not for the distance of the knife from the air passage, he would have thought that he had made an opening into it. He had scarcely said the word, when the young woman cried out that she was dying, and instantaneously dropped down on the floor a lifeless corpse, to which all their efforts could not restore the slightest symptom of animation. This happened in the presence of nearly 400 spectators, and the body was examined next day, in the presence of full as many, with the most rigorous minuteness.—Every part of the body was carefully dissected; but there was not a particle of morbid structure any where to be found. An examination of the heart, however, disclosed the cause of the melancholy catastrophe. The right auricle was distended like a bladder with air, which rushed out when cut open, without any admixture of blood. Fluid blood was found in the other cavities of the heart, as also in the different vessels. Great quantities of air were found in all the vessels. There was no other unnatural appearance in any part of the body.

We have not the smallest doubt, that Dupuytren was perfectly correct in his conclusion, that air had rushed in through one of the veins of the neck, and thus caused instant death. It is, perhaps, the only case on record of the kind, and so unlikely to happen often, that it can be no serious objection to any operation on the neck or other part of the body. We think it is a pathological fact, however, which bears on the physiology of the circulation. It proves that the heart acts as a sucking as well as a forcing pump, otherwise air would never have passed from a cut vein in the neck down into the right chambers of the heart. It is highly probable, that, in consequence of the morbid state of the parts, the mouth of the cut vein had remained patulous, and thus readily admitted the air.

---

8. *Great Tumour removed from the Neck, by Dupuytren.* Our readers are in possession of the facts attendant on the melancholy case of a young woman, operated on by M. Dupuytren, where the introduction of air by a vein caused instant death in the operation room. The case now to be stated, was sent, as a set off to the other, to the Royal Academy of Medicine, and read at the same time. The patient Madame Thiebault, 21 years of age, was a favourite of the Duchess of Berry, and about two years previously perceived a small tumour on the top of the right shoulder, which enlarged to such a size, that M. Boyer was consulted. He advised that no operation should be performed, and prognosticated that the removal of the tumour would be fatal. This advice not being relished, M. Dupuytren was called in. He found an enormous tumour occupying the posterior and lateral part of the neck, on the right side, extending from the mastoid process to the inferior angle of the scapula. The tumour was hard and unequal on its surface. It was every where firmly adherent to the neighbouring parts. The skin covering it was tense, and full of dilated veins. The patient experienced sharp pains in the tumour, and was unable to use the right arm, in which there was a sense of unpleasant formication.

Taking into consideration the age and constitution of the patient, the rapid growth of the tumour, and the inevitable fatality of the disease, if left to itself, M. Dupuytren magnanimously undertook its removal, and the lady as magnanimously submitted to the terrible operation, which was performed on the 27th September, 1822, in the following manner.

M. Dupuytren made an incision, ten or twelve inches in extent, over the greatest diameter of the tumour—that was, from the top of the cervical column to the acromion scapulæ. The trapezius muscle being thus exposed, was divided, and the tumour itself came in view. By a long, painful, and we need hardly say, dexterous dissection, during which many arteries were cut and tied, this enormous tumour was turned out by main force and free incisions. It fell on the ground and bounded like an elastic ball. Several arteries, which gave out blood freely, were secured, and the immense wound was dressed. The intrepid lady never complained during this terrific dissection, but talked calmly to the surgeon all the time. In the night of the same day the lady was seized with pain in the throat, with difficulty of swallowing; but these symptoms were removed by a moderate bleeding. A kindly suppuration became established—granulations shot forth—the limits of the wound decreased—and the lady got perfectly well, and has remained so.

The tumour weighed six French pounds—was of a fibro-cellular texture—and contained some intermixed portions of albuminous lymph. In some points, the tumour appeared to be taking on the carcinomatous character.

The event falsified the prognosis of M. Boyer; but, probably, there were few other surgeons than M. Dupuytren, who would have undertaken so terrible an operation.

---

9. *Purulent Ophthalmia of Infants.** Mr. Ryall, by the most persevering industry, and no inconsiderable talent, has established, for several years past, a National Ophthalmic Infirmary in Dublin, and raised for himself an unquestionable reputation in the line of practice to which he has attached himself. And now, that the main difficulties of his undertaking are overcome, we are glad to see him employ an occasional leisure hour in laying before the profession some of the results of his experience.

The purulent ophthalmia of infants, or acute inflammation of the conjunctiva, consigns to irremediable blindness, by its unfavourable termination, many children annually. The patients brought to the Eye-Institution affected with this disease, are, for the most part, the offspring of the very poorest class—and, many of them, those born in the Lying-in-Hospital. The mothers too often neglect to apply for medical aid till all hope of affording relief is fled. Some do not come till protrusion of the iris has taken place. One woman lately brought, in a wine-glass, both the lenses of the infant's eyes, with part of the vitreous humour which had escaped before application was made for relief. Mr. Ryall is of opinion, notwithstanding authority to the contrary, that the disease in question is communicated to the infant at the time of birth, by some morbid secretion of the mother. However this may be, the power which the ophthalmic discharge afterwards possesses of communicating the ophthalmia, is indisputable. Mr. R. had, at the time of writing, several nurses under his care, in whom the ophthalmia had been

---

* Isaac Ryall. Dublin Transactions, vol. iv.

caused by the accidental application of the discharge from the infants, eyes to their own. Our author is likewise of opinion that the ophthalmia produced by gonorrhœal is more violent than that resulting from leucorrheal matter—and more apt to be attended with excessive tumefaction of the palpebræ—chemosis—profuse and ill-conditioned discharge, with rapid tendency to disorganization. In common ophthalmia, the disease often begins in one eye when it is on the wane in the other, from sympathy—but, in the disease now under consideration, if one eye escaped the first inoculation, and was afterwards carefully guarded from contact with the discharge from the other, it escaped altogether.

This ophthalmia, like gonorrhœa, will, in many instances, yield readily to very simple treatment, nay, will even sometimes spontaneously cease. This lulls to a dangerous security.

"Assuming it as a principle, that the destructive progress of the disease is influenced more by the specific virulence of the inoculating fluid, than by any other cause, and my great object being to subdue its action on the secretory surface, I have an earlier recourse to stimulants than the mere definition of acute inflammation would appear to indicate, or than might be deemed expedient by those who 'consider the discharge merely as a symptom, and that neither the process of restoration is materially affected, nor the organ in any way endangered by its continuance.'

"By this early substitution of the stimulant and mildly astringent for emollient applications, I have known the discharge to have been suppressed, and the progress of the disease consequently arrested, in very many cases which, experience and analogy warrant me in saying, would have otherwise terminated unfavourably, or at best have been protracted for several weeks: indeed, the very character of the inflammation would warn us against the long use of emollients.

"The purulent ophthalmia of infants shews itself generally within four or five days after birth, in redness of the conjunctiva lining the palpebræ, and in a thin discharge, which, if permitted to rest on the cilia, agglutinates them so firmly, as to require some degree of force to separate them; in effecting which, a copious flood of tears gushes forth. These appearances are usually accompanied with great impatience of light, feverish heat, exacerbated in the evenings, and not unfrequently with fits. In a short time the discharge becomes puriform, when there is a remission of the pyrexia, the conjunctiva becomes greatly distended, exhibiting, as has been observed, the appearance of red velvet. The eye-lids are now so much swoln, that in the attempt to examine the eye, the orbiculares muscles are everted, requiring even force to replace them, and mechanical aid to retain them *in situ*. The bowels, if neglected, either remain costive, or discharge mucous, or frothy and greenish stools." 349.

Although the disease will now sometimes cease spontaneously, we have much more reason to dread bad consequences, as the death of the cornea, protrusion of the iris, escape of the humours, sinking of the eye, or, what is worse, troublesome and disgusting staphyloma—all which, in almost every instance, might be prevented by early and proper attention.

"If recourse be had in the early stage of the disease to medical aid, (which, as has been deplored, particularly with the poor, is too seldom the case,) the treatment is then indicated by the definition of 'acute inflammation.' A leech or two, according to the strength of the patient and urgency of the symptoms, are to be applied to the under lid. The eye is to be frequently fomented with a decoction of white poppy heads, which should also be injected between the palpebræ; and, as well to prevent agglutination of the cilia, as to correct the morbid secretion of the tarsi, an ointment, consisting of one part of the ointment of the nitrate of quicksilver, and seven parts of that of spermaceti, should be applied to them twice or thrice a-day. A grain each of the submuriate of mercury and of James's powder should be given every night at bed-time, and a tea-spoon full of castor oil every, or every second morning. The lower extremities, and if fits occur, the whole body, should be daily immerged in a tepid bath; and the apartment in which the patient resides, if it be practicable, well ventilated, since foul air, and crowded situations, are peculiarly favourable to the malignancy, and perhaps to the propagation of the disease." 350.

When the discharge has passed into a more virulent form, becoming profuse and thin, assuming a deep yellow or greenish colour, it is then time to adopt a different mode of treatment. Various collyria have been recommended, and doubtless have proved useful, but our author has found none so efficacious in changing the action of the secretory membrane, and arresting the progress of the disease as a solution of the nitrate of silver (the crystallized nitrate as being more pure than the fused) in proportion of two or three grains of the mineral to the ounce of simple distilled water, frequently and briskly injected between the lids with a silver or ivory syringe. All warm fomentations and cataplasms should now be laid aside; but if tumefaction of the palpebræ continue, a cold cataplasm of alum-curds, confined between two folds of thin linen or muslin, may be applied during the night, or between the periods of using the injection. Should the conjunctiva be greatly extended by effusion, a leech may be applied to the inner surface of the lower lid, or the projecting portions be removed with a pair of scissors. The strength of the solution may be gradually increased to the proportion of eight grains of the nitrate to the ounce of distilled water. The calomel and antimony are to be occasionally administered at night, and a dose of castor oil, infusion of manna, or magnesia, in the morning. Mr. Ryall also recommends a tea-spoon full of oil of turpentine in a little syrup, if the bowels be much deranged. Uuder this treatment, the disease will generally yield in a few days, the discharge gradually assuming a lighter colour and a ropy consistence, till it entirely cease.

10. *Laryngitis—Tracheotomy.** Inflammation about the larynx, and especially about the glottis, performs the work of destruction so quickly, if not arrested, that many lives have been lost for want of the operation now under consideration. Inflammations of this kind, are often too far advanced to be checked by depletion, when the practitioner is called in; but if respiration can be carried on, for a time, we are pretty sure of subduing the original disease, and thus snatching a victim from a most cruel death. Indeed, we cannot conceive a more horrible situation than that of a person whose air-passage is gradually closing up, and who is, consequently, suffering a protracted death by suffocation!

*Case.* Mary Dunne, ætat. 30, admitted into the Whitworth Hospital, 25th October, 1823; had been affected with a troublesome cough, since the latter end of August, which had been increased by renewed exposure to cold. On the 21st October, oppression, difficulty of breathing, dull pain about the region of the larynx, and down to the sternum, were added. No appropriate remedies had been employed. On the evening of admission, the cough was frequent, the expectoration tough and copious, respiration difficult, and attended with a hoarse brazen sound—pulse accelerated. It was found necessary to draw blood from the arm, which gave instant relief, and she passed a tranquil night. 26th. The dyspnœa returned. Repetition of the bleeding did not bring the expected relief. 27th. All things getting worse.—Sound of the cough is like that of croup—the respiration difficult, and obstructed by much viscid mucus—pulse 120—pain in the larynx and sternum. Six leeches to the larynx—an emetic of ipecacuan—pills of calomel and ipecacuan. She was a little relieved by the eeches and emetic. 28th. Worse to-day—eight more leeches—"From the report at noon, it appeared that she felt some temporary alleviation after the leeches; her countenance, however, was quite livid, and expressive of extreme anxiety, with a look of wild despair; her eyes protruded; respiration was nearly impracticable; the extremities, as well as the whole surface, were becoming cold; pulse fluttering and feeble, above 130. She was, however, in perfect possession of her intellect, and implored that something might be done to afford her relief under the extreme urgency of her suffering." It was evident that nothing but an operation would now afford relief. Indeed, we think the knife was resorted to quite late enough.

*Operation.* Mr. Carmichael arrived, and almost immediately proceeded to work, judging that the period of affording relief, even by this measure, was probably past.

We shall give but a very brief description of Mr. Carmichael's operation, as it is now frequently performed, though not without some difficulty. An incision, an inch and a half in length, from the lower edge of the thyroid gland, was made through the integuments, and then be-

* Dr. John Crampton—Mr. Carmichael. Dublin Transactions, v. 4.

tween the long muscles, till the trachea was laid bare. This was happily performed with the loss of only a few drops of blood, "notwithstanding the difficulty which the perpetual motion of the trachea opposes in a person incessantly gasping for breath." Two or three rings of the trachea were then divided, and the opening widened by means of a large pair of sharp-pointed scissors, such as Mr. C. employs for the operation of hare-lip. The patient coughed up a large quantity of viscid phlegm through the opening, and seemed much relieved, the appearance of suffocation being quite removed. Our author feels satisfied that a simple incision into the trachea, and the insertion of a tube, would have been productive of no benefit. The grounds on which he makes this assertion do not appear valid to us—and simply because we have seen the division and insertion succeed in as bad a case, (nay a worse one) than that of Mr. Carmichael's. One positive fact is, therefore, worth fifty negative ones. The slit in the trachea soon accommodates itself to the shape of a tube—and if the tube comes out now and then when the patient is expectorating, it is of no consequence. It can be easily replaced. We make this observation, because we know, from experience, that, in many cases, it would be totally impossible to cut out a lozenge of trachea, as recommended by Mr. Carmichael—where, for instance, the parts are swelled and inflamed from the application of blisters, as is often the case; and where the tube lies an inch deep among working muscles, while the trachea itself is in constant motion. Under such circumstances, we have known a simple slit produce such instantaneous relief that the patient fell fast asleep the moment the opening was made.

The patient did well, and only required great attention in clearing away the ropy mucus from the opening in the trachea. The wound closed in a fortnight, and the cure was complete. The promptitude and dexterity with which Mr. Carmichael performed the operation are very praise-worthy.

We would first remark, that the system of local depletion and counter-irritation pursued in the first instance, when the patient entered the hospital, appears to us to have been very inefficient. No disease requires such decisive depletion, local and general, as laryngitis—and we appeal to the record, whether this decisive depletion was put in execution. From the speedy cure, it is evident that there was nothing more than mere inflammation to contend with here—and we are not without a suspicion that it might have been quelled some days before the operation, by very active treatment.

---

11. *Hæmorrhage into the Bladder.** A gentleman, 73 years of age, had been affected, for many years, with diseased bladder, apparently disease of the prostate gland, and attended with the usual symptoms, frequent inclination to make water, ischuria, and sometimes complete

* Mr. A. Copland Hutchison. Med. Repos. Aug. 1824.

retention, requiring the use of the catheter. About the end of the year 1821, the disease became aggravated, the urine occasionally tinged with blood, and a lithic deposit in his water. The prostate gland, on examination, was found to be much enlarged. On the 26th of February, 1824, he was seized with retention of urine, and much difficulty was experienced in introducing a catheter, when a great deal of urine, mixed with some clots of blood, was drawn off. After this, he was pretty well for a few days, requiring, however, the catheter. On the 2nd March, he was suddenly seized with great pain, and sense of distention, although the urine had only been drawn off a quarter of an hour previously. It was now evident that blood was effused in the bladder. No effort could remove this *per vias naturales*, and Mr. Hutchison proposed to Sir Astley Cooper, to cut into the bladder above the pubes. This was done by Mr. H.——, and the coagula of blood scooped out with a table spoon. The patient went on remarkably well for three days, the water being discharged through a syphon in the wound. It was found, however, on examining the interior of the bladder, with the fingers, after the operation, that there were two fungoid tumours projecting into that organ, one on each side of the urethra, and, from these, it was naturally supposed the blood had come. On the fourth day, his spirits and appetite failed—the wound assumed a bad appearance, and he sunk on the 6th day from the operation.

Cases of this kind are rather rare. It is the first which our author, and the second which Sir Astley Cooper had seen. Leave was not obtained to open the body.

---

12. *Successful Amputation at the Hip-Joint.** We have related an unsuccessful operation of this kind, performed lately in Germany; it is our more grateful task now to record the fortunate termination of a similar operation, performed at Montpellier, by the celebrated Delpech. We hope soon to have the particulars of Sir Astley Cooper's case, which also had a favourable issue, at Guy's Hospital.

The able Professor of Montpellier justly observes, that there is probably no rule which can be laid down for the performance of disarticulation of the hip-joint, because the operation must be modified by the state of the parts rendering such a dangerous and difficult measure necessary. It is, on this account, desirable that, in all cases of this kind, we should have an accurate history of the disease, and the condition of parts, prior to the knife being applied for their removal.

*Case.* Joseph Morel had been brought up to the business of agriculture, and enjoyed good health till the age of fifteen. At this period, and after being exposed to very hard labour for days and nights, in succession, he began to experience wandering pains, at first only slight,

* Professor Delpech. Revue Medicale, Sept. 1824.

in the whole of the right thigh. These gradually increased, and as poverty obliged him to continue his miserable mode of life, the limb swelled, and ultimately terminated in an abscess. This abscess was but the prelude to a series of other abscesses, which became fistulous, and gave issue to several fragments of necrosed and exfoliated bone. For 19 years Morel bore the rigour of his fate, with perfect resignation—suspending his labours when fresh swellings and abscesses took place—and renewing them when the discharge of matter or bone gave a temporary respite to his sufferings. He left all to Nature, and rarely had any surgical assistance. In the course of this long period, these abscesses had multiplied so as to cover almost the whole of the thigh with their sequelæ—cicatrices and fistulæ. In this condition, he was brought to the Hospital, St. Eloi, on the 20th August, 1823, then aged 33 years. He was considerably emaciated—appetite irregular—digestion often deranged—pulse frequent and sharp—chills in the evenings, with fever, succeeded by profuse perspirations in the night. The whole of the thigh was in a state of engorgement, and exceedingly mis-shapen. The *soft* parts were as *hard* as wood—if we may be allowed an Hibernicism. The probe could be passed through each fistulous opening to the bone, which was, in some places naked, and in others carious. Deep incisions into the thigh did not enable the surgeons to ascertain the exact nature of the disease, and a year's residence in the hospital, under various modes of treatment, only shewed an increase of hectic fever and emaciation. It was evident that nothing but amputation could give the poor man a chance of life, and the state of the thigh rendered the removal impracticable, or at least useless, in any other part than at the articulation. This step being determined on, M. Delpech appears to have been under much alarm lest the hæmorrhage should prove too powerful to be overcome in the course of the operation:—he, therefore, determined to cut off the main channel previously to the amputation, by tying the artery at its issue from the abdomen. One reason for this appears to us rather curious—namely, the danger of the assistant stationed to compress the artery, being more attentive to the steps of the operation than to his own immediate duty!

*Operation, 22nd June,* 1824. The patient being properly placed, an incision was made over the inguinal artery, through a mass of condensed parts that caused much difficulty in laying bare the vessels. At length they succeeded in passing a ligature under the artery, and securing it by a double knot, one end of the ligature being cut away. This done, the outline of the internal flap (that was to be) was traced in the integuments, at the upper and inner part of the thigh, with a scalpel—a measure the utility of which we cannot well understand. A large single-edged knife was next plunged in, close to where the artery was tied, and its point directed so as to come out below the hip, and as much externally as possible—passing close to the neck of the femur, on the *inside* and just above the little trochanter, which was avoided by a slight inclination of the edge of the knife inwards. The knife, by cutting its way to the surface, thus formed a flap of conical shape, eight inches in length, and consisting of the whole mass of soft parts, at the upper and inner part of the thigh. During this section, very little blood escaped. The flap was held up by the hands of an assistant, when the neck of the femur and the little trochanter came in view. The thigh being now thrown into a state of abduction, the head of the bone formed a protuberance in its articular capsule, and was readily laid bare, by dividing the latter with a scalpel. This effected, the luxation of the bone instantly followed, with a noise which was easily recognized as the signal of disarticulation. The ligamentum teres was next divided. With the same large knife, a horizontal cut was now made of all the soft parts that remained on a level with the great trochanter, *and from the surface inwards*, finishing this cut at the external edge of the capsular ligament. During this incision, a large abscess was laid open, and on turning up this external flap, a number of tubercles were seen, divided by the knife, and presenting a curious green colour. The wound made by these incisions was of enormous extent, but more regular in surface than might be expected, in consequence of the condensation or, as it were, solidification of the different structures, into one homogeneous mass— a circumstance that rendered the drawing out and tying of the arteries a matter of considerable difficulty.*

When the vessels were secured, M. Delpech experienced another difficulty in approximating the flaps, in consequence of the extreme rigidity of the parts. By great exertion, this difficulty was overcome, and several sutures were inserted, to aid the other dressings in retaining the parts together. Numerous adhesive straps were necessary.

The patient took, immediately after the operation, two grains of opium. The pains continued six hours, after which the patient was pretty tranquil during the day. The night was passed without sleep, the man being harrassed with febrile heat, sense of suffocation, thirst, and pain in his head. 22*nd*. Pulse quick and sharp—face slightly flushed—belly painful towards the hypogastrium. Lavements, fomentations, diluents, five grains of hyosciamus. Two stools, in the course of the day. Night calm, and the patient had some sleep. 23*d*. Pulse much less frequent—patient tranquil, but very weak. Had a good night, and several hours' sleep. 24*th*. Craves for food. The dressings are drenched with a reddish serosity. 26*th*. The dressings, as far as the adhesive straps, were removed. Much pus comes from the centre of the stump. The patient appears to do well, and is allowed light aliment. 29*th*. Another dressing—most of the straps removed—the

* We recollect amputating a boy's thigh, close up to the hip, where long-continued disease of the bone and soft parts had turned the whole of the latter into a substance exactly like brawn. The blood-vessels on the surface of the stump preserved their natural calibre and sphericity, and we apprehended that no ligature would secure them. It was with considerable difficulty we could draw them out, so as to tie them. The boy did well, and never had a bad symptom afterwards.—*Rev.*

wound united in several places—three ligatures came away. *6th July.* The suppurative discharge is much diminished—the ligatures are all come away. From the centre of the wound, a tuberculous mass was this day discharged, followed by a great flow of purulent matter. During the next four days, the patient perspired profusely, when asleep, and had heat of the skin, thirst, and quick pulse. Nevertheless, he did not complain ; had a good appetite ; and rapidly gained strength. His rations were diminished. On the 11th, two other tubercular masses came away, after which the cicatrization of the wound made great progress. *18th.* The night sweats are much less abundant, and all the hectic phenomena are disappearing. The stump is almost healed, and the patient leaves his bed every day. *22nd.* He begins to walk about the ward on crutches. In the beginning of August, the patient was permitted daily to go about the town. On the 20th August, a small exfoliation from the brim of the acetabulum came away from the only point of the stump which remained unclosed. After this, the wound closed, and he was soon able to have an artificial member fitted on, when he took his departure in good health.

In his remarks on this case, M. Delpech reiterates his opinion, that the greatest danger and difficulty would have been encountered, had not the artery been first secured in the groin. We can hardly participate in this opinion. At the same time, we see no particular objection to this precautionary measure, except the additional pain given to the patient, and the increased length of time which takes place during the operation, every minute of which seems an hour to the sufferer.*

* From some cases which occurred in the practice of the translator, many years ago, he is thoroughly persuaded that this operation, in many instance at least, might be greatly simplified, and rendered much less dangerous. A patient had disease of the thigh to such an extent that amputation was obliged to be performed as high as possible, and consequently where no tourniquet could be used. The circular incisions made, the vessels that presented themselves were tied, with little difficulty, and very trifling loss of blood. The bone was bared a couple of inches, and then sawn through. It was found that the saw went close to the little trochanter, and it was evident to the writer, as well as to all present, that the head of the bone could have been scooped out in less than two minutes, and thus the operation at the hip performed, without intending it. The writer has often, since that period, regretted that he did not take out the head of the bone, in order to prove the practicability of it, as he is convinced it would have but very little increased the danger of the operation. He has seen cases where the thigh was carried off by cannon shot, and where the head of the bone might have been easily turned out, in the common way of amputation.

